# Case Report: Digitally driven tooth autotransplantation using surgical templates and three-dimensional printed donor tooth replica

**DOI:** 10.3389/froh.2025.1537468

**Published:** 2025-03-19

**Authors:** Iuliana Babiuc, Marius Ștefan Diaconeasa, Viorel Ștefan Perieanu, Mădălina Adriana Malița, Irina Adriana Beuran, Mihai Burlibașa

**Affiliations:** ^1^ Department of Dental Technology, Faculty of Midwifery and Nursing, Carol Davila University of Medicine and Pharmacy, Bucharest, Romania; ^2^ Dental Essence, Private Practice, Bucharest, Romania

**Keywords:** tooth autotransplantation, surgical template, donor tooth replica, digital dentistry, case report

## Abstract

Tooth autotransplantation is a procedure involving the surgical repositioning of a tooth or dental germ from one site in the mouth to another within the same individual. A successful procedure requires a donor tooth with healthy cementum and periodontium, gentle surgical maneuvers, a well-fitted neoalveolus, and a short extraalveolar time of the donor tooth. Digital technology increases the accuracy and predictability of the tooth autotransplantation procedure. Surgical templates generate a more precise neoalveolus, with good stability of the donor tooth, and decrease the surgical time. Using a donor tooth replica ensures an optimal morphology of the new alveolus, thus reducing the injury of the root and the extraoral time of the donor tooth. This case report presents the tooth autotransplantation technique, that was employed to reposition a maxillary premolar with two fused roots on a mandibular recipient site. Digital planning, two surgical guides (one for each root), and a 3D printed tooth replica were used to ensure good accuracy, prognosis, and reduced treatment time. The procedure is conservative and offers considerable advantages to the patient, such as retaining natural teeth and proprioception.

## Introduction

Autotransplantation is a procedure involving the surgical repositioning of a tooth or dental germ from one site in the mouth to another within the same individual, offering a viable alternative to dental implants and prostheses (1). Tooth autotransplantation was first reported in the 1950s, with initial results demonstrating only a 50% success rate (1). Understanding the biologic foundation of tooth autotransplantation improved the prognosis. Animal studies (2–4) showed that placing a root with healthy cementum in the alveolar bone would lead to the formation of the periodontal ligament, whereas root planning treatments led to resorption or ankylosis (2). It is therefore considered that a healthy root surface plays an important role in the recreation of the periodontal space (5).

Tooth autotransplantation has become an important alternative for replacing missing teeth, with considerable advantages. Unlike implants, the periodontal space of autotransplanted teeth provide proper thermal and proprioception responses, leading to an optimal masticatory function (6, 7). The volume of the alveolar bone is maintained, due to physiological stimulation, which ensures improved esthetics, arch form and dentofacial development in young individuals (5). The treatment cost is generally lower when compared to other therapeutic options, such as dental implants or prosthetic restorations (6). However, the applicability is limited by the availability of suitable donor teeth and recipient site. Potential complications include external root resorption and ankylosis due to periodontal damage, as well as compromised periodontal healing (8). Also, the infraocclusal position and shape of the transplanted tooth may require further procedures (orthodontic or prosthetic) (9).

Successful tooth autotransplantation procedures require a donor tooth with healthy cementum and periodontium, gentle surgical maneuvers, a well-fitted neoalveolus, and an extraalveolar time of less than 15 min of the donor tooth (1). The condition of the donor tooth plays an important role. Highest success rates have been reported in teeth with incomplete rhizogenesis, when the root development is one-half to two-thirds (10). Root formation can continue in the recipient site, ensuring the vitality of the donor tooth. No endodontic treatment is needed, but regular check-ups are recommended (11). When the rhizogeneses is complete, the donor tooth can still be autotransplanted, but root canal treatment is required (12). In this case, it is advisable that the patient is no older than 30 years of age (11). The ideal donor tooth has a simple shape, can be easily extracted, and the recipient bony site should be wide enough to accommodate it (9). Tooth autotransplantation should be avoided in patients with uncontrolled systemic conditions and endocrine disorders that may cause root resorption (11). Smoking seems to have an insignificant effect on the survival rate of autotransplantation after 5 and 10 years (13).

Digital dentistry has enhanced the treatment planning and the accuracy of tooth autotransplantation, thus improving the success rates (14, 15). The use of cone-beam computed tomography (CBCT) in tooth autotransplantation led to higher survival and success rates compared to conventional methods, as it offered precise information regarding the morphology of the donor tooth and of the recipient site (16). The donor tooth replica, used to ideally configure the recipient site, plays a significant role in tooth autotransplantation, as it preserves the donor tooth, reduces significantly its' extraalveolar time and minimizes the injury of the root surface when trying the fit in the new alveolus (17, 18). 3D printed surgical guides generate a more precise neoalveolus, which leads to a good stability of the donor tooth (19, 20). Surgical guides significantly reduce the time of the procedure, and improve treatment outcomes (20–22). A randomized clinical trial showed that fully guided tooth autotransplantation increased the predictability in terms of donor tooth position, establishing ideal conditions for functional recovery and restoration (21).

The use of platelet-rich fibrin (PRF) in tooth autotransplantation procedure enhances soft tissue healing and accelerates recovery (23, 24). PRF may improve bone formation and reduce the risk of ankylosis and bone loss. PRF's ability to promote healing and regeneration of periodontal tissues and pulpal formation plays a crucial role in root development and overall success rates (25). The use of PRF in tooth autotransplantation is documented in case reports and case series, mostly involving donor teeth with incomplete root formation (23–25).

This clinical report describes a guided autotransplantation of a maxillary premolar with complete root formation and fused roots, to a mandibular edentulous site, with the aid of digital tools for planning, two surgical guides for the creation of a new alveolus and a 3D printed tooth replica for an optimal adaptation of the donor tooth to the recipient site. PRF was used to enhance the healing of the extraction site, to promote periodontal regeneration, gingival tissue healing and new bone formation in the recipient site.

## Case report

**Figure 1:**
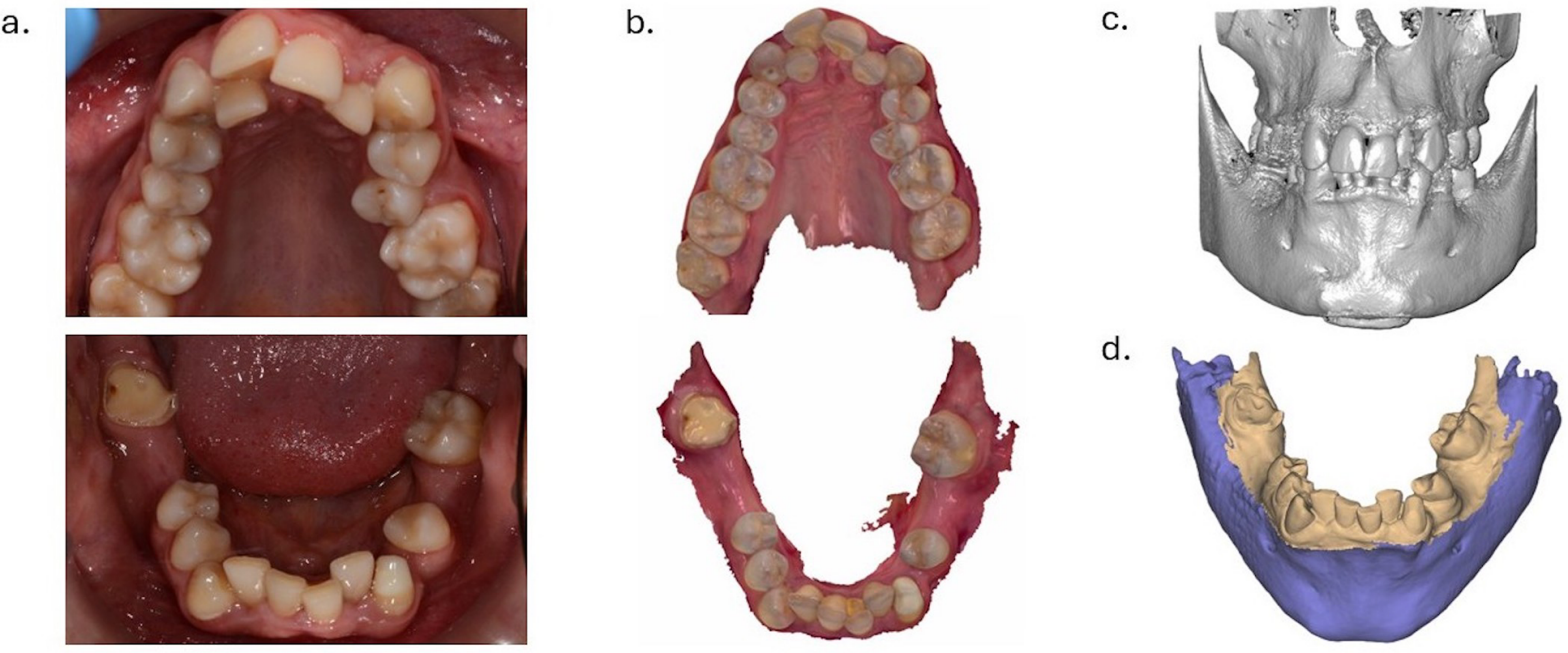
Diagnostic procedures for tooth autotransplantation of a mature tooth in a 27 years old male patient—acquiring clinical, intraoral scan and cone beam computed tomography data to create the virtual patient. **(a)** Preoperative occlusal aspects of the maxillary and mandibular arch. **(b)** Intraoral scan. **(c)** Cone beam computed tomography examination. d. Virtual patient obtained by the superimposition of the intraoral scan file over the CBCT file.

A 27 years old male patient, with no relevant medical history, non-smoker, presented with the request to completely rehabilitate his dental status. Clinical and radiological examination indicated multiple carious lesions and unrestorable roots, as well as severe crowding of the maxillary dental arch (Figure 1a). Patient presented a high plaque index, suggesting inefficient dental hygiene habits.

Initial treatment consisted in extractions of unrestorable teeth, dental fillings and root canal treatments. Efforts were made to improve the level of oral hygiene. After the initial treatment, an approach to solve the maxillary crowding and the mandibular edentulous spaces was explored. Autotransplantation of the second upper left premolar on a right mandibular first molar recipient site followed by orthodontic treatment was considered a valid treatment option.

**Figure 2:**
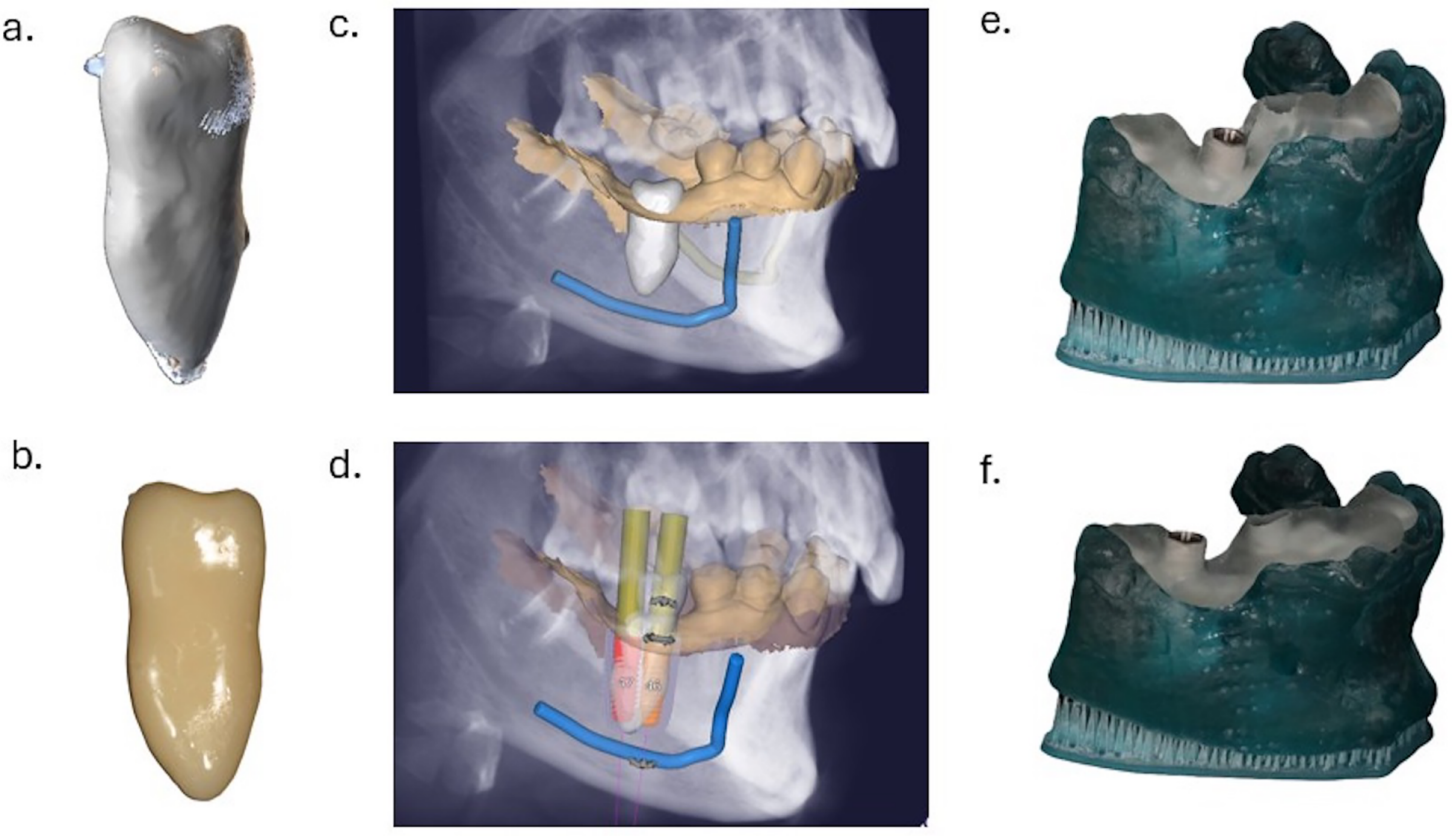
Digital planning for the autotransplantation procedure: digital segmentation of the donor tooth and the fabrication of a three-dimensional printed donor tooth replica; the digital file of the donor tooth was later placed digitally in the recipient site and two surgical guides following each of the donor tooth's roots were designed and printed. **(a)** Donor tooth segmentation from the CBCT file using BlueSkyPlan software. **(b)** 3D printed donor tooth replica. **(c)** Digital planning of the donor tooth position in the mandibular arch using Exocad software. **(d)** Planning osteotomy with two surgical guides for the neoalveolus. Since the donor tooth displayed two fused roots, two separate surgical guides were designed for each root. **(e)** Surgical guide for anterior root. **(f)** Surgical guide for posterior root.

**Figure 3:**
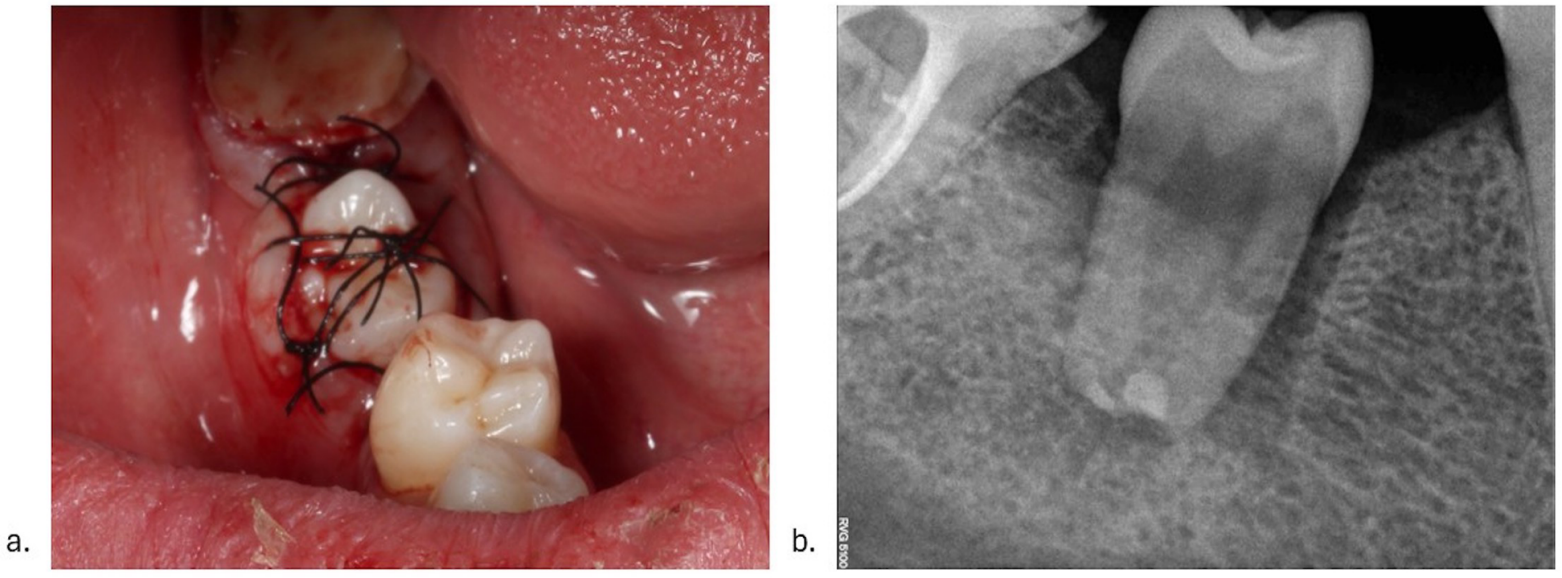
Intraoral aspect following tooth autotransplantation of tooth 25 in the position of 46. **(a)** Postoperative view of the autotransplanted tooth. Suture stabilization was acquired. **(b)** Postoperative radiograph of the autotransplanted tooth, showcasing a loose fit.

Clinical examination and cone-beam computed tomography (CBCT) analysis indicated a good-sized edentulous space of the first mandibular molar, and a favorable morphology of the donor tooth, with two merged roots. The intraoral scan (3Shape) (Figure 1b) was superimposed over the CBCT file (Planmeca) (Figure 1c) to create the virtual patient (Figure 1d). The digital segmentation of the tooth to be transplanted from the CBCT scan was performed (BlueSkyPlan) (Figure 2a). The autotransplantation procedure of the donor tooth was planned digitally (ExoCad), by superimposing the 3D file of the donor tooth over the virtual mandibular arch (Figure 2c). Two surgical guides were designed (Figure 2d) and 3D printed (V Print Splint Clear, Voco), following the direction of each root of the premolar (Figures 2e,f). A three-dimensional replica of the donor tooth was 3D printed (NextDent C&B MFH, NextDent), to optimize the adaptation of the dental root to the neoalveolus before tooth extraction (Figure 2b).

**Figure 4:**
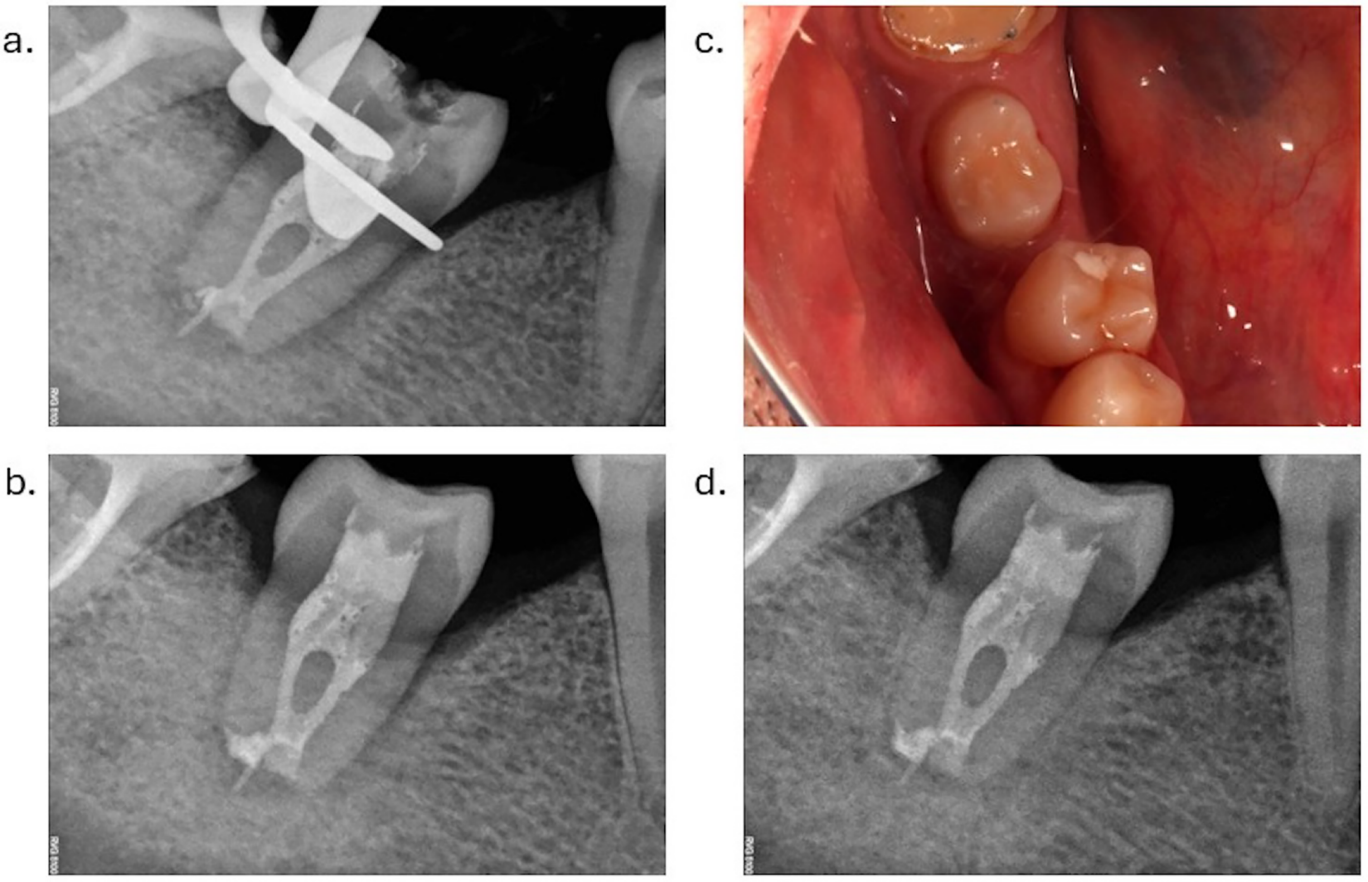
Tooth monitoring following autotransplantation procedure for 10 months. **(a)** Radiograph with endodontic treatment, one month after autotransplantation. Reorganization of the periodontal space can be observed. **(b)** Radiograph three months after autotransplantation, with visible narrowing of the periodontal space. **(c)** Occlusal view of the donor tooth ten months after autotransplantation. **(d)** Radiograph ten months after autotransplantation, with a normal width of the periodontal space.

During the clinical appointment, after initial anesthesia, a muco-periosteal flap was raised, and the edentulous alveolar bone of lower right first molar was exposed. The new alveolus was prepared using the surgical guides and the corresponding drill kit (SKY pro guide surgical tray, Bredent). Additional drilling under abundant saline cooling was necessary to optimally adapt the replica of the donor tooth to the new socket. The upper left second premolar was later extracted as atraumatically as possible. Platelet-Rich Fibrin (PRF) plugs were placed in the extraction socket, followed by sutures. The tooth was held using the plier, avoiding contact with the root surface. Apical resection was performed under abundant saline cooling, followed by retrograde mineral trioxide aggregate root canal filling (MTA, Angelus). The premolar was then positioned in the neoalveolus, and PRF liquid was injected around the donor tooth. PRF membranes were positioned around the tooth, to seal the newly created periodontal space from the oral environment. Sutures were placed around and on top of the premolar to ensure a proper wound closure (Figure 3). No additional splinting was used to allow a functional stimulation of the periodontal space. One month after the autotransplantation, the endodontic treatment was performed (Figure 4a).

The patient was monitored clinically and radiographically. The mobility of the root decreased progressively and was within normal limits after 3 months (Figure 4b). Radiographs showed the reorganization of the periodontal space, with a normal aspect at 10 months follow-up (Figures 4c,d). CBCT examination taken one year after the procedure confirmed the success of the autotransplantation. Orthodontic treatment was initiated (Table 1).

**Table 1:** Timeline with the relevant stages of the treatment of guided tooth autotransplantation.

Treatment sequence	Findings/treatment
Initial examination Panoramic x-ray	Multiple decays, failing teeth Class II Angle malocclusion Crowded teeth in the maxillary arch
Treatment of active pathology	Professional cleaning and education for proper hygiene Extraction of unrestorable teeth (18,38,36,34,45,46) Endodontic treatment of teeth 13,32,33,35,47 Restorative treatment of decayed teeth
Treatment planning for tooth autotransplantation	CBCT (Planmeca), intraoral scan (3Shape), virtual patient (Exocad) Digital tooth segmentation of 25 (BlueSkyPlan) Planning the position of the donor tooth in the mandibular edentulous site (Exocad) 3D printed donor tooth replica (Asiga, NextDent) Creation of 2 surgical guides (Asiga, Voco), one for each root, of the transplanted premolar
Guided tooth autotransplantation	Creation of a neoalveolus with surgical guides (Bredent surgical kit). Perfecting the osteotomy to better fit the donor tooth replica Atraumatic extraction of the donor tooth Apical resection and MTA retrograde filling Positioning the donor tooth in the recipient site Applying PRF in the extraction socket and in the recipient site Sutures to stabilize the donor tooth Control x-ray
Follow-up	2 weeks—suture removal 1 month—root canal treatment, control x-ray 3 months—reduced tooth mobility, no signs of inflammation, control x-ray 10 months—complete formation of the periodontal space, normal mobility, control x-ray 12 months—no symptoms, CBCT shows a normal aspect of the periodontal space, orthodontic treatment starts

## Discussion

Autotransplantation has become a valuable alternative to consider when replacing missing teeth, with important benefits for the patient, such as retaining natural teeth and proprioception. Patient selection plays a significant role in the success of tooth autotransplantation. In the present case, the male patient was young, non-smoker, with no general pathology. A good-sized edentulous crest and a favorable morphology of the fully developed donor tooth, with no periodontal and carious pathology, indicated the procedure. Efforts were made to reduce the plaque index of the patient, to ensure a good periodontal space regeneration.

Digital dentistry added important tools to improve the success rate of tooth autotransplantation (14, 15). Advances in digital tomography and rapid prototyping have been shown to reduce the technique sensitivity and increase the predictability of the procedure (16, 17). Virtual planning of the transplantation, 3D prototyping and 3D printed surgical guides ensure an accurate socket preparation and of the position of the donor tooth (13). In comparison to the classical approach, these digital applications allow for a reduced extra-alveolar time of the donor tooth, minimized trauma of the root surface, and improved treatment outcomes (17, 20, 21). In our case, a loose fit of the donor tooth to the bony walls was achieved, which does not apply extensive pressure on the root surface.

PRF was used to enhance the healing of the extraction site and the integration of the donor tooth in the neoalveolus. Studies suggest that PRF is effective in promoting the success of tooth autotransplantation (24) by enhancing periodontal regeneration (25), improving gingival tissue healing and increasing new bone formation in extraction sockets (26, 27).

Conventional sutures were preferred to splinting, allowing a physiological mobility during the healing period, which is thought to stimulate an optimal regeneration of the periodontal space (20). A good morphology of the neoalveolus, acquired using the surgical templates, also contributed to a loose fit of the donor tooth and a good stability.

After ten months, the donor tooth showed normal clinical and radiologic parameters, with physiological mobility and no signs of periodontal inflammation.

This case report aims to increase the knowledge on guided tooth autotransplantation. The level of evidence regarding this technique is relatively scarce. More research and published cases are needed to build sufficient evidence for practical and theoretical applications. A randomized clinical trial (21) on guided tooth autotransplantation showed increased predictability in terms of donor tooth position, in comparison to the conventional method. However, this study had limited statistical power given the sample size (*N* = 14) and other limitations regarding the methods employed for optimal positioning of the donor tooth.

In conclusion, digital applications enhance the precision and predictability of tooth autotransplantation. CBCT analysis and the use of a 3D printed replica ensures good adaptation of the donor tooth to the new alveolus, reduces the extraalveolar time of the donor tooth and the injury of the root surface during the procedure. Surgical guides have been shown to generate a more precise neoalveolus, with good stability and a more appropriate position of the donor tooth. PRF enhances the healing of the extraction site and the integration of the donor tooth in the neoalveolus, by improving gingival tissue healing and promoting periodontal regeneration and alveolar bone formation.

## Data Availability

The datasets presented in this article are not readily available. Requests to access the datasets should be directed to dental.essence2010@gmail.com.
